# Advances and Limitations in Electroanatomic Mapping for Infant Catheter Ablation

**DOI:** 10.19102/icrm.2019.100602

**Published:** 2019-06-15

**Authors:** Philip M. Chang

**Affiliations:** ^1^Congenital Heart Center, University of Florida Heath, Gainesville, FL, USA

**Keywords:** Catheter ablation, electroanatomic mapping, pediatric, tachyarrhythmia

## Abstract

Advances in electroanatomic mapping (EAM) technology have facilitated improved success and safety profiles in the field of catheter ablation. However, these advances in their current iteration may be of limited value in ablation performed in very small children. The present case report highlights the application of current EAM technologies in an infant with incessant arrhythmias and includes a discussion regarding the application and limitations of newer mapping and ablation technologies in this unique and fragile patient group.

## Introduction

**Figure 1:**
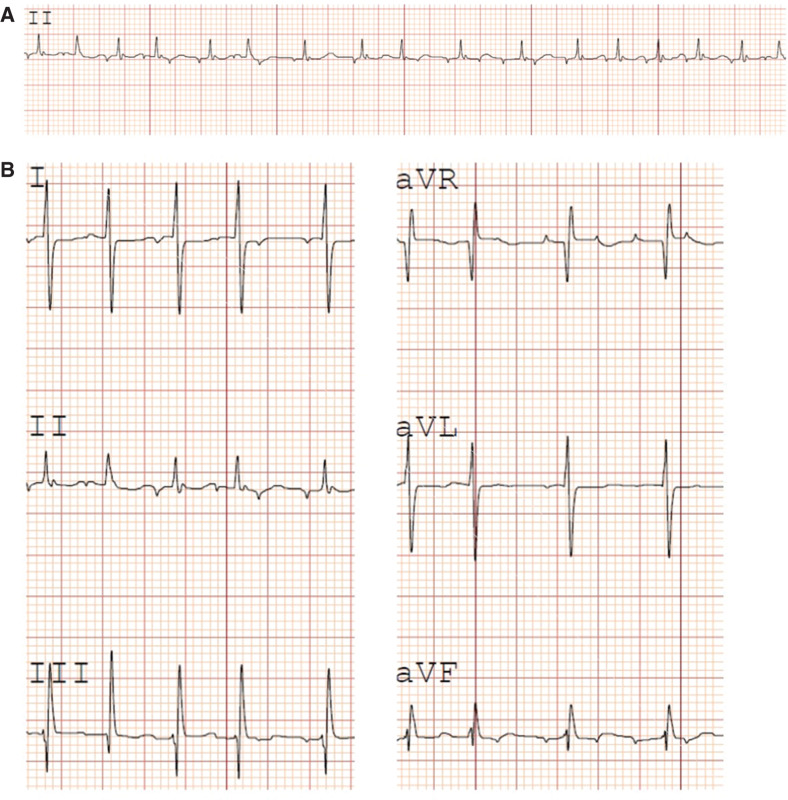
Electrocardiogram tracings of focal ectopic atrial tachycardia. **A:** Lead II rhythm strip demonstrates focal ectopic atrial tachycardia with variable atrioventricular conduction. **B:** Limb lead tracings demonstrating focal ectopic P-wave morphology.

A three-month-old, 4.2-kg infant (full term, birth weight: 3.5 kg) was brought to the electrophysiology (EP) laboratory for EP testing and catheter ablation of prenatally diagnosed and postnatally recalcitrant atrial tachycardia. A large secundum atrial septal defect (ASD) was also prenatally diagnosed and postnatally confirmed. Focal ectopic atrial tachycardia was documented on 12-lead electrocardiogram **(Figure 1)**. Hospital course was notable for circulatory compromise and respiratory distress requiring intubation and invasive vascular line insertion. Additional complicating events included recurrent bloody stools (concerning for necrotizing enterocolitis, gut hypoperfusion during incessant tachycardia, or dietary allergies), aspiration and ventilator-associated pneumonias, and urinary tract infection. This also made enteral medication administration difficult and inconsistent. Several antiarrhythmic medications were trialed, including enteral digoxin, propranolol, flecainide, and sotalol, while enteral; intravenous amiodarone; intravenous esmolol; and, additionally, heavy sedation (including dexmedetomidine) were also implemented. However, rhythm control was never achieved despite the use of combination therapies. Furthermore, the observation of an extremely wide QRS tachycardia while on flecainide and digoxin required their cessation. Sotalol uptitration was prevented due to QT prolongation. Although rate control could be achieved with combination intravenous amiodarone and esmolol, multiple attempts at conversion to enteral dosing while preserving stable hemodynamic and respiratory statuses were unsuccessful.

Electrophysiology testing and catheter ablation were performed with bilateral femoral venous access [ie, 7-French (Fr) catheter in the right femoral vein and 5-Fr catheter in the left femoral vein] and transesophageal catheter insertion. Vascular access was difficult given the concomitant presence of a right lower-extremity peripherally inserted central catheter (PICC) line, left femoral arterial line, and right internal jugular central venous catheter. The Ensite™ Precision™ system (Abbott Laboratories, Chicago, IL, USA) was incorporated for electroanatomic mapping (EAM) support. A 4-Fr quadripolar catheter was used for initial EAM geometry creation and a 4-Fr deflectable decapolar catheter was then cannulated in the coronary sinus. For activation mapping and ablation, the quadripolar catheter was exchanged with a 7-Fr small-curl, 4-mm nonirrigated-tip radiofrequency (RF) ablation catheter. Fluoroscopy was required during vascular access and catheter advancement/manipulation to avoid entanglement with existing vascular lines, to confirm right versus left atrial positioning during mapping, and to monitor catheter course and stability during ablation. Anticoagulation with serial doses of intravenous heparin was maintained.

**Figure 2:**
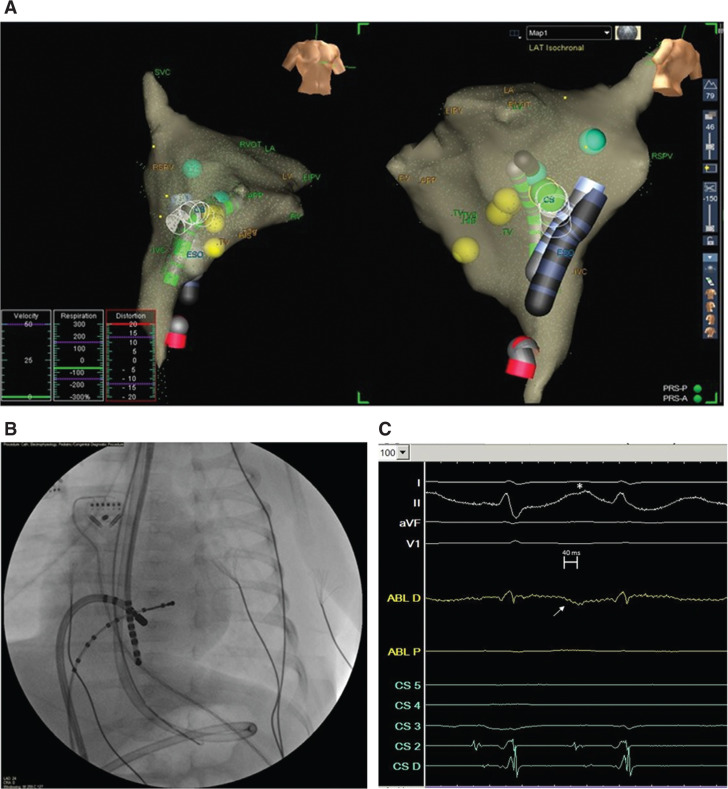
Electroanatomic and fluoroscopic images of catheter locations and local ablation signals at the site of ablation success. **A:** EAM images in standard anteroposterior and modified lateral projections showing the decapolar coronary sinus catheter, transesophageal catheter, and ablation catheter (in the inferior vena cava). Green dots indicate the sites of catheter ablation. **B:** Fluoroscopic left anterior oblique projection of the heart with reference catheters and ablation catheter at the site of ablation success. **C:** Local ablation catheter electrogram signal at the site of successful ablation. Distal ablation (ABL D) tracing and lead II ECG tracing are gained up for visualization purposes. The local atrial signal onset at ABL D (arrow) was 40 ms earlier than the onset of the surface P-wave (asterisk).

While simultaneous multipoint acquisition could be used for initial geometry creation, point-by-point contact activation mapping was performed instead herein with the ablation catheter. Use of a multielectrode mapping catheter that would have permitted simultaneous, multipoint mapping was deferred due to vascular access constraints and considerations. Mapping involved a cumulative time of 30 minutes for acquisition and manual timing evaluation, with a total of 49 points included. Focal atrial tachycardia was present, with the site of earliest activation localized to the left atrial aspect of the inferior, anterior rim of the ASD **(Figures 2A and 2B)**. Ablation with a 20-second, 20-W maximum power delivery resulted in arrhythmia suppression during RF application, with subsequent resumption. The catheter tip was adjusted slightly superiorly and a 20-second RF application, this time with a 30-W maximum power delivery, resulted in tachycardia termination in six seconds without further recurrence **(Figure 2C)**. A 20-second insurance lesion was also delivered. Postablation testing demonstrated normal atrioventricular conduction, whereas ventricular pacing using the ablation catheter confirmed midline, decremental conduction. The catheters and sheaths were removed at the conclusion of the procedure.

## Discussion

This case represents one of the smallest pediatric patients who has undergone mapping and ablation using the most current iteration of one of the most widely used EAM systems for pediatric and adult electrophysiology procedures. While a reasonable outcome without complications was achieved, the case serves to highlight the progress made as well as the continued limitations inherent in the application of advanced mapping systems for ablative therapy in very small patients.

Electroanatomic mapping systems were developed to facilitate arrhythmia characterization with regard to both mechanism and location and to ensure a better accuracy of ablation delivery. Technology has since progressed to these systems providing real-time visualization of multiple catheters within the cardiac and intravascular spaces; sophisticated cardiac geometry creation with the integration of intracardiac echocardiogram, computed tomography, or magnetic resonance imaging data; dense and rapid mapping of complex arrhythmias within heterogeneous myocardial substrates; real-time monitoring of catheter-tip pressure during ablation delivery; and visualization tools permitting the performance of procedures with very little or no fluoroscopy.^1–5^ Fundamentally, the most widely available and employed EAM systems rely on impedance- and/or magnetic field–based tracking to determine and follow catheter electrode locations. Signal acquisition is typically performed with contact between the catheter electrode(s) and myocardial tissue to record either unipolar or bipolar electrograms. The acquired signals can then be interpreted with regard to their timing (in relation to a prespecified reference site) and amplitude. Timing and amplitude provide the framework for both activation and substrate mapping within the geometric structure created during the movement of catheters within the heart.

New and advanced features of EAM systems now permit rapid, automated, multipoint acquisition for faster and simultaneous geometric shell creation, substrate definition (ie, scar), and arrhythmia characterization.^6–8^ In addition, advances in ablation technology now permit more accurate local electrogram signal acquisition during ablation delivery, focused open irrigation directed to the site of tip-to-tissue contact during RF energy delivery, and extrapolation of lesion efficacy based on duration and force application data.

Despite many of these advances and features being theoretically at our disposal in this current case, we found that most were of limited utility in such a small patient. Furthermore, the use of these advanced features and tools, particularly in conjunction with the recommended proprietary catheters, would have introduced additional and potential procedural risks as well as substantial increases in procedural costs.

Data on catheter ablation procedures and outcomes in very small patients have generally been favorable. Blaufox et al. on behalf of the Pediatric Catheter Ablation Registry (PCAR) noted comparable success and complication rates between patients aged 18 months or younger and their older pediatric counterparts.^9^ The authors additionally found that more infant ablation procedures were performed by centers that submitted more procedures overall to the PCAR, which suggests that procedural volume and experience may contribute to these comparable outcomes between age groups. Akdeniz et al. reported on their single-center experience with catheter ablation in very small infants aged younger than one year, with a total of five patients having undergone successful ablation.^10^ In this small cohort, two had notable congenital heart disease (CHD) and all had accessory pathway–mediated supraventricular tachycardia that was ablated with cryotherapy or RF without major complications. EAM was employed in all elective cases (one case was performed emergently without EAM). Bigelow et al. reported two cases of successful accessory pathway ablation with EAM in infants with CHD and Wolff–Parkinson–White syndrome.^11^ Ozaki et al. compared outcomes of RF ablation in patients weighing less than 10 kg to those of larger patients.^12^ Consistent with PCAR data findings, more patients weighing less than 10 kg had CHD as compared with larger patients (45%). Between two and four vascular access sites were employed and catheters of sizes of up to 8.5 Fr were used. RF ablation and EAM were used in all cases with acute success achieved in approximately 91% of patients, with a 15% recurrence rate. Finally, Takeshita et al. reported successful RF ablation of monomorphic right ventricular outflow ventricular tachycardia in a 2.9-kg infant with Ebstein’s anomaly via single catheter access through the right internal jugular vein.^13^

**Figure 3:**
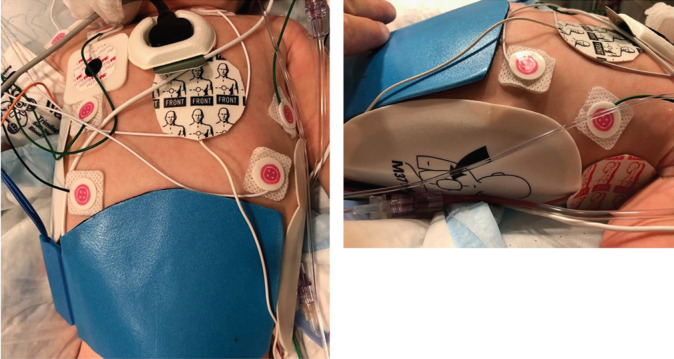
Preprocedural chest and abdominal preparation with electrocardiogram electrodes, impedance field patches, RF grounding pad, and defibrillation patches.

Despite the technological advancements that have occurred, however, multiple limitations remain, particularly when applying advanced EAM systems in infant ablation procedures. First, the procedural setup remains rather cumbersome, with a substantial amount of hardware and patches that need to be applied on an extremely limited body surface area **(Figure 3)**. Patch overlap and poor skin contact can lead to suboptimal or misleading data acquisition or problems with EAM functionality during the procedure. Furthermore, many infants undergoing ablation may be critically ill and frequently have associated invasive vascular access and monitoring tools, as was true in the presented case, which further reduces the available space for positioning and applying EAM hardware.

Second, the choice and availability of vascular access remains a dominant variable in the planning and performance of infant ablation procedures. Catheter-based procedures in small patients and particularly in those weighing less than 10 kg are heavily impacted by the availability and size of vascular structures. In general, a “less is better” strategy is favored, wherein the smallest/minimum amount of access is used in order to reduce the risk of vascular complications and ensure the highest likelihood of preserving access sites for future procedures. Preexisting or previously inserted vascular lines can also limit or eliminate sites for access and must be accounted for during preprocedural planning. In our patient’s case, internal jugular and lower-extremity venous access were already in active use. Femoral arterial sheath access also distorted anatomical landmarks, making ipsilateral femoral venous access more challenging.

Third, newer EAM technology generally requires the application of specific catheters in order to make use of advanced features for mapping and ablation. In our presented case, while a flexible, small-Fr-sized catheter was used for geometry creation with simultaneous, multipoint acquisition, activation mapping was performed in a traditional point-by-point scheme. The open platform of the Ensite™ Precision™ system (Abbott Laboratories, Chicago, IL, USA) permits the use of any catheter for EAM; however, more sophisticated mapping features require the positioning of a reference catheter and a second proprietary magnetic-field-based, sensor-enabled, multielectrode catheter for roving, automated, and multipoint acquisition. This unfortunately also generally necessitates that larger vascular access be available. Proprietary catheters are also often substantially stiffer, thereby increasing the risk of mechanical trauma or perforation in the infant heart.

Fourth, although fluoroscopy-free procedures can be performed in many pediatric procedures, the use of fluoroscopy was considered necessary in our case; specifically, the confirmation of appropriate vascular access, avoidance of the entanglement of catheters and wires with the patient’s PICC line, and verification of catheter location in the right or left heart across the ASD all required fluoroscopy. We also encountered some difficulties with EAM distortion, requiring further fluoroscopy to confirm catheter position and for map correction. This distortion may have been related to the limitations seen with field scaling in such a small patient with potentially variable and changing impedance fields during the course of the procedure, as may occur during the course of ventilator changes and shifts in intravascular fluid status.

Current RF catheters that employ force-sensing technology are presently only available in irrigated-tip catheter form. Given the size and thickness of the infant heart, irrigation-assisted RF ablation is unnecessary. Furthermore, although it can be argued that force-sensing would be helpful in infant ablation to ensure appropriate applied contact along thin-walled structures or in close proximity to vulnerable structures, this feature alone may not justify the substantially higher cost of these catheters in comparison with standard, nonirrigated models. Although force-sensing catheters could be used without irrigation, this may increase the risk of luminal clot formation or inadvertent air embolus introduction. Further systematic evaluation of the use of force-sensing ablation catheters in infant ablation procedures is needed to better understand their role, safety, efficacy, and cost impact.

## Conclusion

Significant advances have been and continue to be made with EAM systems and their integration in both common and complex mapping and ablation procedures. Many features have limited applicability in infant ablation, owing to vascular access and size limitations, the risk of myocardial trauma or perforation, and higher procedural costs without associated clear advantages over conventional mapping and ablation tools and techniques. Successful ablation in the presented case was achieved with a careful selection of catheters, the selective integration of available tools during EAM, and the application of standard principles of activation mapping and RF ablation. The development of complimentary technology that permits the integration of newer EAM features specifically in smaller patients could potentially permit earlier ablative therapy in this unique subgroup of patients, thereby avoiding significant morbidity, both cardiac and noncardiovascular organ dysfunction, medication toxicity, and even mortality in the setting of recalcitrant or incessant tachyarrhythmias.
